# Assessment of Post-operative Complications in Patients Undergoing Thyroid Surgery in a Tertiary Care Hospital in Eastern India

**DOI:** 10.7759/cureus.42549

**Published:** 2023-07-27

**Authors:** Tejaswi Mishra, Ipsa Mohapatra, Varsha Srivastava, Tapas K Rout

**Affiliations:** 1 Department of General Surgery, Maharaja Krushna Chandra Gajapati (MKCG) Medical College, Berhampur, IND; 2 Department of Community Medicine, Kalinga Institute of Medical Sciences, Bhubaneswar, IND

**Keywords:** family history, dysphagia, risk factors, complications, thyroidectomy

## Abstract

Background

Thyroid surgeries, among the most common surgical procedures globally, present with varied complications. This study is aimed at identifying the complications and selected variables associated with thyroid surgery.

Methods

In this cross-sectional, retrospective, record-based study, a total of 107 patients who underwent thyroidectomies and satisfied the inclusion criteria, were recruited using convenience sampling technique. This study was conducted at a tertiary care hospital from January 2021 to December 2021. Data were collected from medical records using a researcher-created data extraction form after ethical approval from the institutional ethics committee. The data were analyzed using Epi Info software (Atlanta, GA: Centers for Disease Control and Prevention), with a p-value of <0.05 considered to be statistically significant.

Results

Of the 107 patients who underwent thyroidectomies, 92 (85.9%) reported one or more complications. Complications were most common (90.2% of patients) in the 25-34 years age group and among females (83.3%). The most common complications were dysphagia (30.84% of patients), voice change (21.50%), and respiratory obstruction (8.41%). Temporary hypocalcemia developed in 3.74% of these patients, while tracheal injury and hematoma were documented in 3.74% and 1.87%, respectively. Tobacco users (14.9%), alcohol users (16.8%), those eating a non-vegetarian diet (61.9%), and those eating saturated fats (13.0%) suffered more complications. Family history (p=0.03) was found to be significantly associated with complications.

Conclusion

The most common post-thyroidectomy complications in this group of patients were dysphagia and voice change, while hypocalcemia, tracheal injury, and hematoma were rare complications. Tobacco users and alcohol users reported more complications. Complications were more common in those with a family history of thyroid disease and those who were underweight.

## Introduction

Thyroid diseases are among the most commonly reported endocrine problems. The thyroid is a hormone-producing gland controlling the body’s metabolism [1]. According to research conducted in India, 42 million people are thought to suffer from thyroid disease [2]. Over the past decade, numerous regional studies have indicated an increase in thyroid diseases in India [3]. Medical or surgical treatment options are available to manage thyroid diseases. Thyroidectomy is one of the most frequent medical procedures in this regard, and it can be either partial or complete [4]. The procedure is becoming more feasible due to advancements in diagnostic techniques and surgical safety.

With advancements in treatment and surgeons’ expertise, post-operative morbidity and mortality rates are declining [5]. However, the major side effects of thyroid surgery can include persistent hypoparathyroidism, hypocalcemia, post-operative hemorrhage, and recurrent laryngeal nerve damage [6,7]. Some studies have investigated the risk factors of thyroidectomies, identifying factors such as age, sex, and thyroid disease type [8,9].

There are no publications reporting the same results or findings similar to those from this region of the Indian state of Odisha, according to a review of the most recent medical literature on thyroid surgery and associated complications [5-9]. Against this background, this study is intended to identify the complications and selected variables associated with thyroidectomies.

## Materials and methods

**Figure 1:**
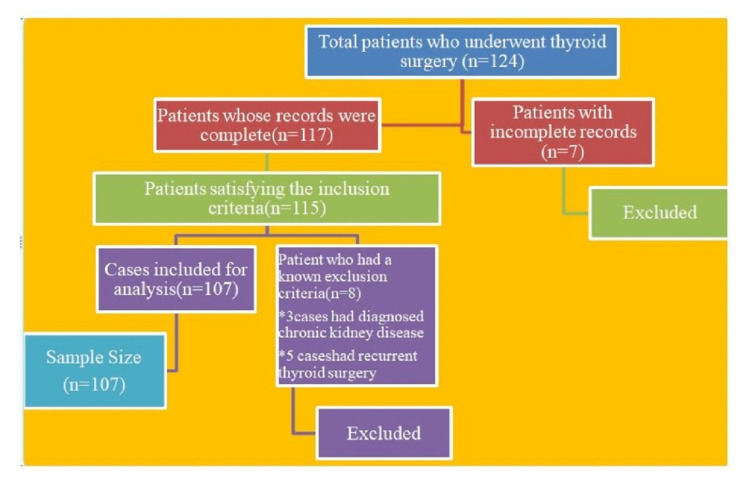
Sample recruitment with sampling frame and final sample size.

A cross-sectional, records-based retrospective study was conducted among 107 post-operative thyroid surgery patients in the general surgery department of the selected tertiary care hospital from January 2021 to December 2021. During the study period, 107 of 124 patients operated for thyroid issues satisfied the inclusion criteria and were included in the final sample (Figure 1).

The convenience sampling technique was used. Data were manually extracted from the patients’ medical records, using a researcher-created extraction form. All information was related to risk factors for post-thyroidectomy complications (e.g., age, sex, and type of thyroid surgery, time taken for the first consultation with the doctor and where did they go {health-seeking behavior}), and associated complications (e.g., recurrent laryngeal nerve injury, tracheal injury, hoarseness, hematoma formation, hypocalcemia, wound infection, loss of high-pitched sound, seroma, and thoracic duct injury). The complications documented in the records were noted, along with the post-operative findings based on clinical assessments. The biochemical and/or radiological investigations that had been documented were also noted. As calcium level is routinely measured both pre-operatively and post-operatively (for both total and subtotal thyroidectomies), the findings in this regard were extracted from the case sheets and noted.

All thyroid surgery cases with complete medical records and proper documentation were included in this study. The exclusion criteria were chronic kidney disease, pre-operative hypoparathyroidism, and a history of dysphonia and recurrent thyroid surgery.

Operational definitions

Operative morbidity/complications of thyroid surgery are as follows: temporary or permanent recurrent laryngeal nerve, temporary or permanent hypoparathyroidism, incision site infections, bleeding, hematoma, flap edema, seroma, superior laryngeal nerve injury, systemic complications, and organ injury.

Statistical analysis

The collected data were entered into a Microsoft Excel spreadsheet and analyzed using Epi Info software (version EnUS 7.4.2.1) (Atlanta, GA: Centers for Disease Control and Prevention). All categorical variables were expressed as frequency and proportion. Descriptive statistics were expressed as mean±SD, with range. A chi-square test and Fisher’s exact test were used as tests of association, with <0.05 considered to be statistically significant.

## Results

**Figure 2:**
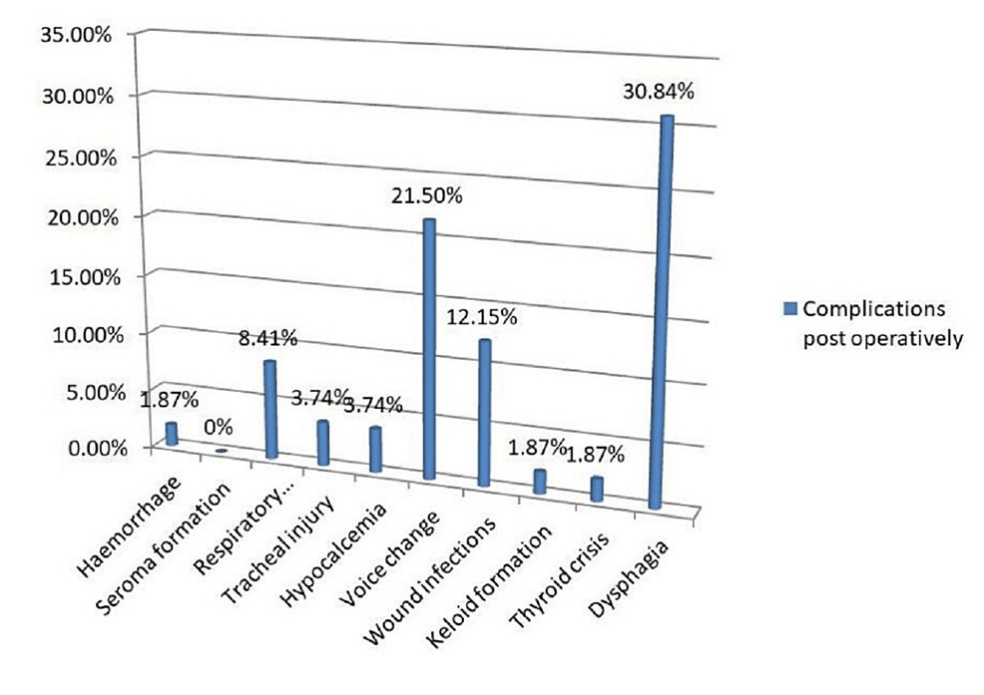
Frequency of post-thyroidectomy complications.

This study reviewed the case records of 107 patients who underwent thyroidectomies between January 2021 and December 2021 (Table 1). The prevalence of complications was found in 85.9% of patients. Females (67.2%) outnumbered males. Participants’ age ranged from 18.0 to 64.0 years, with a mean age of 33.6±9.7 years. The mean BMI was 25.7±3.8. Out of 107 patients, 56.0% had normal BMI, 28.9% were overweight, 11.2% were obese, and 3.7% were underweight. In total, 57.9% of participants lived in rural areas, while 45.7% were unemployed. Of 107 participants, 92 (85.9%) reported one or more complications (Figure 2).

**Table 1:** Association of socio-demographic characteristics of study participants with complications (n=107). *The values were obtained using chi-square test for association. **The values were obtained using Fisher's exact test for association.

Variable		Complications present (n=92)	Complications absent (n=15)	p-Value
Age group (years)	18-24 (n=18)	13 (72.3%)	5 (27.7%)	0.14^*^
	25-34 (n=41)	37 (90.2%)	4 (9.8%)	0.46^*^
	35-44 (n=24)	21 (87.5%)	3 (12.5%)	0.92^**^
	45-54 (n=15)	13 (86.6%)	2 (13.3%)	0.75^**^
	55-64 (n=9)	8 (88.8%)	1 (11.2%)	0.81^**^
Gender	Male (n=35)	32 (91.4%)	3 (8.6%)	0.41^*^
	Female (n=72)	60 (83.3%)	12 (16.7%)	
BMI	Underweight (n=4)	4 (100%)	0	0.92^**^
	Normal (n=60)	52 (86.7%)	8 (13.3%)	1.0^*^
	Overweight (n=31)	27 (87.1%)	4 (12.9%)	0.92^*^
	Obese (n=12)	9 (75%)	3 (25%)	0.47^**^
Permanent residence	Urban (n=45)	37 (82.2%)	8 (17.8%)	0.50^*^
	Rural (n=62)	55 (88.7%)	7 (11.3%)	
Educational status	Primary (n=11)	10 (90.9%)	1 (9.1%)	1.0^*^
	High school (n=27)	26	1	0.14^**^
	Intermediate (n=30)	23 (76.6%)	7 (23.4%)	0.16^*^
	Graduate (n=28)	24 (85.7%)	4 (14.3%)	0.79^*^
	Postgraduate (n=11)	9 (81.8%)	2 (18.2%)	1.0^**^
Employment status	Unemployed (n=49)	43 (87.8%)	6 (12.2%)	0.84^*^
	Employed (n=58)	50 (86.2%)	8 (13.8%)	
Type of family	Nuclear family (n=44)	36 (81.8%)	8 (18.2%)	0.45^*^
	Joint family (n=50)	44 (88%)	6 (12%)	0.78^*^
	Three-generation family (n=13)	12 (92.3%)	1 (7.7%)	0.78**
Marital status	Unmarried (n=37)	29 (78.4%)	8 (21.6%)	0.18^*^
	Married (n=70)	63 (90%)	7 (10%)	

The most common complications were dysphagia (30.84% of patients), temporary voice change (21.50%), and wound infections (12.15%). Hypocalcemia and tracheal injury were reported in 3.74% of patients, while 30.84% had either a recurrent laryngeal nerve injury or difficulty in swallowing and/or speaking. Hemorrhage, keloid formation, and thyroid crisis were reported in 1.87% of patients.

**Figure 3:**
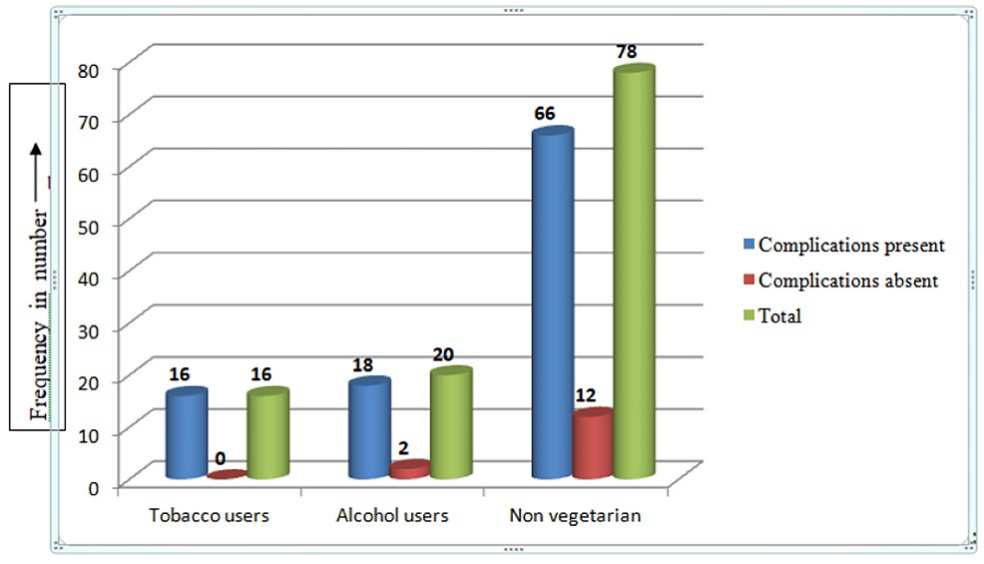
Risk factors among the study participants (n=107).

Tobacco users (14.9%), alcoholics (16.8%), and those eating a non-vegetarian diet (61.9%) experienced more complications. All patients who used tobacco experienced complications after surgery, as did 90% of patients who consumed alcohol. Altogether 84.6% of patients who were not vegetarians had complications (Figure 3).

Health-seeking behaviors (e.g., who they first approached about their symptoms and when) did not show a statistically significant association with post-operative complications. However, patients who displayed such behaviors within one month had comparatively fewer complications than those who displayed such behaviors after more than one month. In this study, it was determined that patients who first visited a tertiary hospital for the management of their symptoms had fewer complications than those who visited a local doctor or primary healthcare center (PHC) (Table 2).

**Table 2:** Complication status with the health-seeking behavior (n=107). *The values were obtained using chi-square test for association. **The values were obtained using Fisher's exact test for association. PHC: primary healthcare center

Health-seeking behavior		Complications present	Complications absent	p-Value
Delay	>10 days (n=9)	8 (88.9%)	1 (11.1%)	0.81^*^
	10 days-1 month (n=76)	64 (84.2%)	12 (15.8%)	0.60^*^
	>1 month (n=22)	20 (90.9%)	2 (9.1%)	0.69^**^
Whom did you first approach	Local doctor (n=18)	17 (94.4%)	1 (5.6%)	0.44**
	PHC (n=30)	26 (86.7%)	4 (13.3%)	0.86**
	Tertiary hospital (n=59)	49 (83.1%)	10 (16.9%)	0.49^*^

**Figure 4:**
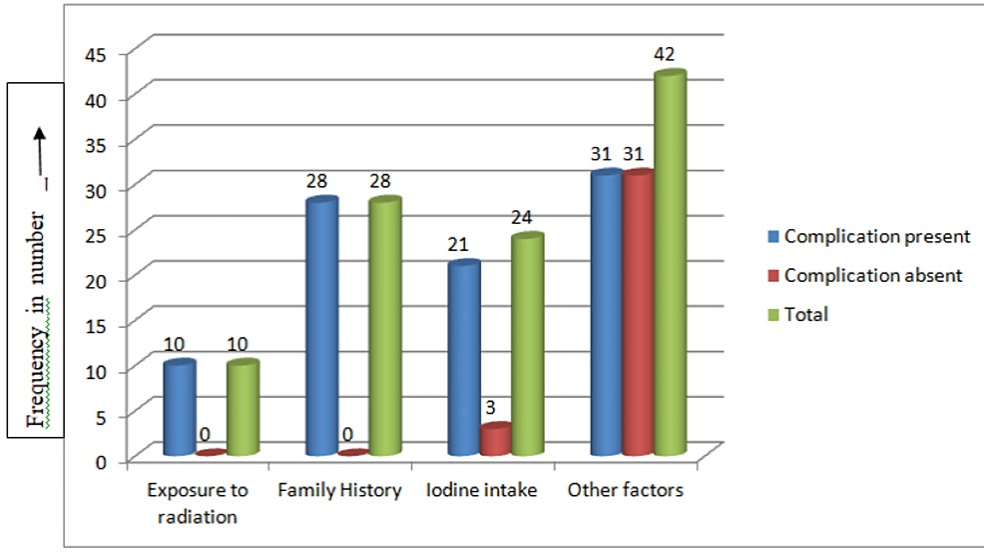
Clinical risk factors with complications after surgery (n=107).

All patients with a family history of thyroid disease (28 patients) and those who had been exposed to radiation (10 patients) experienced complications after the surgery. Among the patients with iodine deficiency as a risk factor, 75% experienced post-operative complications, while 39.2% of patients had other risk factors (e.g., age, gender, or being overweight) (Figure 4).

Patients could be divided as follows based on their blood group: 46.7% of patients were type O+, 22.4% were type A+, 21.4% were type B+, 4.6% were type AB+, 2.8% were type A-, and 1.8% were type B-. The diagnosis was benign in 73.83% of patients and malignant in 26.17%.

In total, 9.3% of participants had been diagnosed with papillary carcinoma of thyroid and were experiencing post-operative complications. Of 41.1% of participants who had been diagnosed with colloid goiter, 75% experienced complications after surgery. All 15.8% of patients diagnosed with follicular thyroid experienced complications after surgery, while 91.3% of 21.4% of patients diagnosed with multinodular goiter experienced complications. Altogether 11.2% of patients were diagnosed with a solitary thyroid nodule, 83.3% of whom had complications after surgery. Finally, 0.009% of patients were diagnosed with thyroid isthmus nodules.

Of 107 patients, 27.1% had total thyroidectomies, and they all experienced side effects, such as bleeding, seroma development, respiratory injuries, hypocalcemia, and voice changes. In total, 70% underwent hemithyroidectomies, of which 81.3% experienced complications; 18.6% of patients have undergone an isthmusectomy and 0.93% have undergone total thyroidectomy plus modified radical neck dissection (Table 3).

**Table 3:** Association of types of surgery performed with complications. *The values were obtained using Fisher's exact test for association. **The values were obtained using chi-square test for association.

Management	Complication present (n=92)	Complication absent (n=15)	p-Value
Total thyroidectomy (n=29, 27.1%)	29 (100%)	0 (0%)	0.02*
Hemithyroidectomy (n=75, 70%)	61 (81.3%)	14 (18.7%)	0.06**
Isthmusectomy (n=2, 18.6%)	1 (50%)	1 (50%)	0.64*
Total thyroidectomy + MRND (n=1, 0.93%)	1 (100%)	0 (0%)	0.29*

## Discussion

Females outnumbered males in the current study. However, although more females underwent thyroidectomies, complications were more common among the male participants. Alqahtani et al. found that in Saudi Arabia, females reported more complications than males among all patients who had undergone thyroidectomies [10]. A 2013 study by Pandey et al. found that 70 out of 80 patients were females [11]. A 2021 study by Alqahtani et al. found that 151 of 182 patients were females [12]. This difference could be due to the operational categorization of the complications. While the current study investigated group of complications, the former study mentioned only hypocalcemia, change of voice, and hematoma [10]. In this study, it was shown that gender, age, and BMI did not have a statistically significant association with post-operative complications. Similar findings were made in a study conducted in Saudi Arabia among patients who had undergone thyroidectomies.

Participants in this study who consumed tobacco and alcohol and followed a non-vegetarian diet experienced more complications, although no statistically significant association was found. All the thyroid disease patients who had been exposed to radiation reported complications after the surgery. Patients with iodine deficiency as a risk factor also had post-thyroidectomy complications. A family history of thyroid disease had a statistically significant association with post-operative complications.

Health-seeking behavior did not have any significant association with post-operative complications. However, patients who displayed this behavior within one month had comparatively fewer complications than those who displayed it after more than a month. This study found that those who first visited a tertiary hospital for the management of their symptoms had fewer complications than those who visited a local doctor or PHC. No similar studies reporting the health-seeking behavior of patients could be found.

Post-operative dysphagia is common after thyroid surgery. In the present study, post-operative dysphagia was the most frequent complication, followed by temporary voice change and wound infections. Hypocalcemia, hemorrhage, and respiratory obstruction were rarely observed. Total thyroidectomies were significantly associated with the risk of post-operative complications. In a 2021 study among 182 patients by Alqahtani et al., 116 patients developed post-operative temporary hypocalcemia, and three patients developed persistent hypocalcemia [12].

As the study was a single-center study, the results may not be generalizable to the state’s whole population. Being records-based, the study also had some limitations. For example, the duration of the surgery and the experience of the operating team could not be related to the complications.

## Conclusions

Dysphagia was the most frequent post-thyroidectomy complication, followed by voice change; whereas hypocalcemia, tracheal injury, and hematoma were rare complications. Males reported more complications. Tobacco usage, alcohol intake, and non-vegetarian diet were associated with higher complications post-thyroidectomy. A decreased incidence of problems was observed in those who sought medical attention promptly and seek for tertiary care hospitals. Those with radiation exposure, family history, and iodine deficiency reported more complications. The complication was more in cases of total thyroidectomy.

The study highlights some of the associated risk factors, like male sex, tobacco, and alcohol use, and those consuming a non-vegetarian diet being more prone to complications. Early consultation and management at a tertiary care setup gave patients a better advantage in terms of lesser complications. The finding of more complications in those with radiation exposure, family history, and iodine deficiency can help surgeons identify the at-risk group of developing complications, who can be better taken care of to avoid complications.

The study highlights some of the associated risk factors among a group of population from the eastern part of India, adding to the evidence pool. The modifiable risk factors can be communicated to the population to avoid the risks involved and avert complications.
